# The Risk of Secondary Knee Procedures After Anterior Cruciate Ligament Reconstruction—A Nationwide Retrospective Cohort Study

**DOI:** 10.3390/jcm14113823

**Published:** 2025-05-29

**Authors:** Han-Kook Yoon, Chang-Min Lee, Hyun-Cheol Oh, Taemi Youk, Sang-Hoon Park

**Affiliations:** 1 Department of Orthopaedic Surgery, National Health Insurance Service Ilsan Hospital, Goyang 10444, Republic of Korea; hangugy@nhimc.or.kr (H.-K.Y.); changmini713@naver.com (C.-M.L.); hyuncoh@nhimc.or.kr (H.-C.O.); 2 Research Institute, National Health Insurance Service Ilsan Hospital, Goyang 10444, Republic of Korea; tmyouk@nhimc.or.kr

**Keywords:** anterior cruciate ligament, big data, knee

## Abstract

**Objectives:** Some following up patients have poor clinical outcomes when they experience anterior cruciate ligament reconstruction. The patient will undergo progression of knee joint osteoarthritis or several secondary knee procedures such as high tibial osteotomy, total knee arthroplasty, menisectomy, meniscus repair, or meniscus transplantation on their knees after anterior cruciate ligament reconstruction. This may be related to the remaining instability of the knee joint, changes in the knee joint biomechanics, and progression of osteoarthritis. This study aims to determine which secondary knee procedures are performed over time after anterior cruciate ligament reconstruction. **Methods:** The National Health Insurance Service-Health Screening database analyzed 146,122 patients who underwent ACL reconstruction surgery between 1 January 2002 and 31 December 2021. Secondary knee procedures were investigated by categorizing them into revisional reconstruction, high tibial osteotomy, total knee arthroplasty, menisectomy, meniscus repair, and meniscus transplantation, respectively. Multivariable Cox Proportional Hazard model analysis was used. The significant predictors for complications (*p* < 0.05) were as follows. **Results:** Among a total of 146,122 patients with anterior cruciate ligament reconstruction, 1073 (0.7%) patients underwent HTO, 908 (0.6%) patients underwent TKA, 15,218 (10.4%) patients underwent meniscectomy, 7169 (4.9%) patients underwent meniscus repair, and 938 (0.6%) patients underwent meniscus transplantation. The hazard ratio differed according to graft type, gender, and age group. **Conclusions:** Patients who undergo ACL reconstruction may experience poor clinical outcomes, such as progression of osteoarthritis and undergoing secondary knee procedures several years after ACL reconstruction. It is important for decision-making, ongoing monitoring, and follow-up care for patients undergoing ACL reconstruction.

## 1. Introduction

The anterior cruciate ligament is the most frequently injured ligament in the knee that needs surgical treatment [1,2]. There are studies about the incidence of ACL injury and reconstruction surgery worldwide. For example, in the United States, studies reported that the annual incidence of ACL tears was 68.6 per 100,000 people, between the years of 1990 and 2010. The rate of ACL reconstruction increased significantly over time in all age groups [3]. Additionally, in Italy, the incidence rate of ACL reconstruction was 21.70 to 33.60 per 100,000 people, between the years of 2001 and 2015. The number of ACL reconstruction procedures increased annually [4]. Additionally, a study on South Korean populations reported that the incidence of ACL reconstruction was 36.4 per 100,000 people in 2008 and increased to 46.4 per 100,000 people in 2016 [5].

However, to our knowledge, there has been no report on secondary knee procedures after anterior cruciate ligament reconstruction. As the number of people performing anterior cruciate ligament reconstruction increases, understanding the natural course of the patient and management after surgery also increases. Some patients undergoing ACL reconstruction undergo several secondary knee procedures, such as revisional reconstruction, high tibial osteotomy, total knee arthroplasty, meniscectomy, meniscus repair, or meniscus transplantation over time.

Analysis of National Health Insurance (NHI) data can provide optimal information on epidemiological patterns of such procedures.

This study aims to determine which secondary knee procedures are performed over time after anterior cruciate ligament reconstruction.

## 2. Methods

This study is a retrospective observational cohort study using extensive nationwide data provided by the National Health Insurance Service (National Health Insurance Service-HealthScreening; NHIS-HealS) with the approval of the institutional review committee of the Ethics Committee of Ilsan Hospital.

Among the patients who were diagnosed with ACL injury (diagnostic codes: S83.50, S83.52, M23.53, M23.63, M23.83, M23.93, M23.01, M23.11, M23.21, M23.31, M23.41, S83.7), patients who underwent ACL reconstruction (N0880, N0881) from 1 January 2003 to 31 December 2021 were included in the study. Additionally, after ACL reconstruction, secondary knee procedures were categorized to revision (N0880, N0881), high tibial osteotomy (N0304), total knee arthroplasty (N2072, N2077), menisectomy (N0821, N0826, N0822, N0827), meniscus repair (N0823, N0828, N0824, N0829) and meniscus transplantation (N0825, N0820).

We also evaluated and compared the hazard ratio (HR) of secondary knee procedures by graft type of reconstruction (autograft vs. allograft), gender (male vs. female), and age (<20, 20–39, 40–49, 50–59, ≥60).

SAS 9.4 (SAS Inc., Cary, NC, USA) was used for all analyses. Multivariable Cox Proportional Hazard model analysis was used for eight independent variables of postoperative complications. In the case of complications and hospital-related variables, there were no missing data due to the nature of the claim data. Cases in which patient-related variables were missing due to information errors were extremely rare, and these were excluded. HR and 95% confidence interval (CI) are presented. The level of significance was maintained with a *p* value of < 0.05.

## 3. Results

From 2003 to 2021, a total of 146,122 patients who underwent ACL reconstruction were found. Table 1 shows the total number of ACL reconstruction cases and the graft used.

Table 2 shows which secondary knee procedure was done after ACL reconstruction. The results show that 1073 (0.7%) patients underwent HTO, 908 (0.6%) patients underwent TKA, 15,218 (10.4%) patients underwent menisectomy, 7169 (4.9%) patients underwent meniscus repair, and 938 (0.6%) patients underwent meniscus transplantation, respectively.

Table 3 shows the hazard ratio of each secondary knee procedure according to type of graft, gender, and age. Due to the number of Autograft-used TKA cases being small, it does not show suitable results.

Table 4 shows the follow-up time. The index date was the first start date of medical treatment for anterior cruciate rupture or anterior cruciate ligament reconstruction, and the follow-up was conducted for up to 19 years from 2003 to 2021.

Table 5 shows the survival time. Survival time means from the start date of the first medical treatment related to the anterior cruciate ligament to the date of secondary surgical treatment. Table 4 and Table 5 in this study provided median and quartile follow-up times.

At first, we examined the difference according to the difference in the graft. Autograft shows lower HR than Allograft for all secondary knee procedures except meniscus transplantation. Each HR was 0.633 (range 0.522–0.767, 95% C.I.) in HTO, 0.817 (range 0.782–0.854, 95% C.I.) in menisectomy, 0.839 (range 0.789–0.892, 95% C.I.) in meniscus repair, and 1.051 (range 0.897–1.233, 95% C.I.) in meniscus transplantation. As seen from the result, meniscus transplantation did not have significant results.

Next, in terms of gender, females show a higher HR of HTO—2.081 (range 1.834–2.361, 95% C.I.); menisectomy—1.232 (range 1.185–1.280, 95% C.I.); a lower HR of meniscus repair—0.927 (range 0.871–0.987, 95% C.I.); and meniscus transplantation also did not have a significant result—0.857 (range 0.715–1.028, 95% C.I.).

Finally, comparing the HR according to age based on those under 20 s, HTO shows higher HR in all age groups (20–39, 40–49, 50–59, >=60). Meniscectomy shows lower HR in all age groups except 50–59, 1.029 (range 0.970–1.092, 95% C.I.), meniscus repair shows lower HR in all age groups, and meniscus transplantation shows lower HR in 50–59, >=60 groups.

## 4. Discussion

The current study provides valuable insights into the incidence and risk factors for secondary knee procedures after ACL reconstruction. Through this study, we found that the risk of secondary knee surgery increases after ACL reconstruction. Considering each risk, it was found that additional knee damage occurred or arthritis progressed.

ACL reconstruction does not entirely restore the functional level of the knee before damage. Remained instability is one of the most crucial complications after ACL reconstruction. Emily et al. reported that an average increase in anterior laxity of 2.7 ± 2.3 mm between the day of surgery and 7 years was significant (*p* < 0.001) [6]. Scott Tashman et al. reported that ACL reconstructed knees were more externally rotated on average by 3.8 +/− 2.3 degrees across all subjects and time points (*p* = 0.0011) [7].

Also, changes in natural knee biomechanism and progression of osteoarthritis may occur after ACL injury and reconstruction. Burak Akesen et al. showed elevated TNF-alpha, collagenase, and TIMP-1 levels due to a ruptured ACL [8]. Timothy W. Tourville et al. reported that ACL-injured patients with abnormal tibiofemoral joint space width had diminished quality of life, increased pain, and increased type II collagen urine CTX-II/serum CPII ratios compared with healthy controls [9]. Urine CTX-II concentrations were significantly higher in ACL reconstruction than the normal control group 16 weeks post-operative, and a high urine CTX-II level was related to knee pain and altered knee function [10].

Our study mainly focuses on the biomechanical explanation of the second procedure after ACL reconstruction. It is thought that this reason can be found in the recently reported ligament biology, especially in the recent development of epithelial membrane theory [11]. Based on our study, epithelial membrane tissue has a role in healing and joint degeneration after injury, and based on this, it provides insight into the cellular and extracellular matrix response of the epithelium after new ACL injury for ligament healing and remodeling—not later big data studies—and it is thought that specific studies on whether it affects joint instability and osteoarthritis progression after reconstruction are needed.

Short- and medium-term clinical outcomes after anterior cruciate ligament reconstruction are usually found to be good. Studies have shown that approximately 80–100% of patients reported normal or near-normal outcomes after surgery [12,13,14,15]. In particular, in the functional evaluation, the Lysholm score improved from 64 before surgery to an average of 92 after surgery, showing normal and similar functional recovery results. However, there are subtle differences when compared to normal knees. In the evaluation of the anterior stability of the joint, the measurement using KT-2000 showed a slight increase in the mean anterior potential at a short follow-up of 2.1 ± 1.4 mm, and 2.8 ± 1.8 mm at a medium follow-up, but it was still acceptable [12]. However, these figures indicate a complete difference from normal knees.

In a study comparing the clinical outcomes of reconstruction following acute and chronic anterior cruciate ligament injury, there were no significant differences between the two groups in mean quadriceps atrophy, Tegner activity score, Lachman test, IKDC score, etc. This suggests that the time between the time of injury and the time of surgery may not have a significant impact on clinical outcomes [6,7,12].

It is known that arthritis is likely to occur in the long term after ACLR [15]. Long-term follow-up revealed that ACL reconstruction does not completely change the natural course of post-traumatic arthritis (PTOA). At 14 years after ACL reconstruction, approximately 57% of patients developed knee osteoarthritis, whereas only 18% of the opposite normal knee developed arthritis. In addition, the prevalence of radiological knee osteoarthritis after ACL reconstruction tends to increase over time, with 11.3% at 5 years after surgery, 20.6% at 10 years, and 51.6% at 20 years [15]. However, compared to patients who underwent reconstruction but did not undergo reconstruction, the relative risk of developing arthritis (RR) in patients who underwent reconstruction was as low as 3.62 (RR in patients who did not undergo reconstruction was 4.98). This suggests that reconstruction does not completely prevent the development of arthritis but has the effect of delaying it to some extent [15].

Post-traumatic OA is the other most crucial complication after ACL injury and reconstruction. May Arna Risberg et al. reported that the prevalence of radiographic tibiofemoral and patellofemoral OA at the 20-year follow-up was 42% and 21%, respectively [16].

Up to 80% of the patients showed radiographic markers of knee OA, such as joint space narrowing and osteophyte formation, after ACL reconstruction within 15 years. Almost up to 50% of those patients with radiographic markers of OA also have symptoms such as stiffness, swelling, and pain [17,18,19,20,21].

These problems lead some patients to undergo secondary knee procedures. Among the secondary knee procedures, the frequency of the procedure was particularly high for the meniscus injury. There can be some contributing factors, such as abnormal loading on knee joints because of altered knee biomechanisms [10,16], there could be pre-existing meniscus injury [22], or improper surgical techniques.

These findings suggest that patients and clinicians should know the potential need for additional knee procedures and consider the risk factors in their decision-making process. For example, older patients or those with high BMI may need more cautious follow-up and rehabilitation—such as reduced body weight—after ACL reconstruction to prevent or delay the need for HTO or TKA [14,23,24]. In addition, there are studies that show differences in the possibility of reoperation of the anterior cruciate ligament according to the graft choice in reconstruction of the anterior cruciate ligament, but few studies have investigated the possibility of secondary surgery other than reoperation [13]. So, the choice of graft type and surgical technique may also affect the risk of secondary knee procedures, and further research is needed to investigate these factors.

Next, for hazard ratio, autograft reveals lower HR for all secondary knee procedures. This may be related to the autograft having a lower failure rate and incidence of complications than the allograft [25,26].

For gender, females reveal higher HR for HTO and menisectomy; however, males reveal higher HR for meniscus repair. We need more research and investigation to obtain this result.

For age, the younger age group (under 20) reveals higher HR for menisectomy and meniscus repair and lower HR for HTO than the older age group.

It may relate to younger individuals being more active than older individuals, which could increase their risk for knee injuries. Also, the biomechanics of the knee joint may differ between younger and older individuals, which could affect the likelihood of developing specific knee injuries. For example, younger individuals may be more prone to ACL tears due to factors such as ligament laxity or muscle recovery after surgery [27,28].

On the other hand, HTO is related to the degeneration of knee cartilage and joints; it can be explained that the older age group reveals a much higher HR of HTO than the younger age group.

Since this study was based on the claimed code data of the National Health Insurance Service (National Health Insurance Service-HealthScreening; NHIS-HealS), it was estimated that a lot of data or codes may be missing in the processing of each hospital. Due to the nature of big data research, the details of each patient cannot be grasped at all, and there is a problem that can only be viewed through a registered code.

There are a number of risk factors that can affect the results of this study, but this study shows that it is shown as a variable by graft type, gender, and age.

The patient’s data include those who underwent surgery between 2003 and 2021, so the follow-up period is not constant. Therefore, some procedures’ hazard ratios were inaccurate, and the trend was only checked. Furthermore, there is a lack of studies comparing regular patients who did not undergo primary ACL reconstruction.

There could be remaining instability or changes in the previous knee joint biomechanisms that can occur after ACL reconstruction. The accelerating progression of osteoarthritis may also occur. The results of our study show that after anterior cruciate ligament reconstruction, several secondary knee procedures such as HTO, TKA, menisectomy, meniscus repair, and meniscus transplantation will be necessary for the knee joint. The incidence of the procedures can be shown as a variable by graft type, gender, and age.

Another limitation of this study was that no mention is made of the possible consequences of opting for conservative management in cases where ACL reconstruction is clinically indicated. Since it is classified by the billing code, it is possible to classify more accurately when it comes to parts related to surgery, but it is difficult to distinguish between the type, duration, posterior tibial slope, and rehabilitation of conservative treatment. Therefore, it seems difficult to add content related to conservative treatment in the current study.

The study results also have important implications for both patients and healthcare providers. Patients considering ACL reconstruction should be informed about the potential risks of secondary knee procedures, and healthcare providers should consider the risk of secondary knee procedures when deciding whether to recommend ACL reconstruction as a treatment option.

The study also highlights the importance of ongoing monitoring and follow-up care for patients undergoing ACL reconstruction. Regular check-ups and monitoring of knee function can help identify potential problems early on, which may reduce the need for secondary knee procedures.

## 5. Conclusions

This study provides valuable information about the risks of secondary knee procedures after ACL reconstruction. Patients who undergo ACL reconstruction may experience poor clinical outcomes, such as progression of osteoarthritis and undergoing secondary knee procedures several years after ACL reconstruction. The findings underscore the importance of informed decision-making and ongoing monitoring and follow-up care for patients undergoing ACL reconstruction.

## Figures and Tables

**Table 1:** Total number of ACL reconstruction cases and the demographic factors.

Total 146,122		Allograft		Autograft		*p*-Value *
		N	%	N	%	
Sex	Male	94,935	78.4%	21,086	84.40%	<0.001
	Female	26,213	21.6%	3888	15.60%	
Age	under 20	15,006	12.4%	3715	14.90%	<0.001
	20–39	60,901	50.3%	14,775	59.20%	
	40–49	24,911	20.6%	4430	17.70%	
	50–59	15,786	13.0%	1773	7.10%	
	over 60	4544	3.8%	284	1.10%	
Revision ACLR		11,102	9.2%	2171	8.7%	0.018

* The level of significance was maintained with a *p* value of < 0.05.

**Table 2:** The secondary knee procedures data after ACL reconstruction.

		Total	HTO After ACLR			TKA After ACLR			Meniscus After ACLR			Meniscus Repair After ACLR			Meniscus Transplantation After ACLR		
			N	(%)	*p*-Value *	N	(%)	*p*-Value *	N	(%)	*p*-Value *	N	(%)	*p*-Value *	N	(%)	*p*-Value *
Sex	Male	116,021	638	0.5%	<0.0001	360	0.3%	<0.0001	11,577	10.0%	<0.0001	5958	5.1%	<0.0001	797	0.7%	<0.0001
	Female	30,101	435	1.4%		548	1.8%		3641	12.1%		1211	4.0%		141	0.5%	
Age	under 20	18,718	18	0.1%	<0.0001	-	0.0%	<0.0001	2225	11.9%	<0.0001	1806	9.6%	<0.0001	121	0.6%	<0.0001
	20–39	75,676	300	0.4%		15	0.0%		7212	9.5%		4130	5.5%		608	0.8%	
	40–49	29,341	379	1.3%		162	0.6%		3190	10.9%		838	2.9%		182	0.6%	
	50–59	17,559	330	1.9%		446	2.5%		2190	12.5%		336	1.9%		26	0.1%	
	over 60	4828	46	1.0%		285	5.9%		401	8.3%		59	1.2%		1	0.0%	

* The level of significance was maintained with a *p* value of < 0.05.

**Table 3:** Hazard ratio of reoperation after anterior cruciate ligament reconstruction due to risk factors.

Risk Factors		HTO After ACLR			Menisectomy After ACLR			Meniscus Repair After ACLR			Meniscus Transplantation After ACLR		
		HR *	95% C.I.		HR *	95% C.I.		HR *	95% C.I.		HR *	95% C.I.	
			Lower	Upper		Lower	Upper		Lower	Upper		Lower	Upper
Graft	Autograft	1.000			1.000			1.000			1.000		
	Allograft	1.581	1.304	1.916	1.223	1.171	1.279	1.192	1.121	1.267	0.951	0.811	1.115
Sex	Male	1.000			1.000			1.000			1.000		
	Female	2.081	1.834	2.361	1.232	1.185	1.280	0.927	0.871	0.987	0.857	0.715	1.028
Age	under 20	1.000			1.000			1.000			1.000		
	20–39	4.337	2.694	6.981	0.791	0.754	0.830	0.534	0.505	0.564	1.210	0.995	1.472
	40–49	11.795	7.351	18.927	0.838	0.794	0.884	0.261	0.241	0.284	0.919	0.730	1.157
	50–59	17.695	11.004	28.454	1.029	0.970	1.092	0.190	0.169	0.213	0.238	0.155	0.363
	over 60	10.802	6.259	18.644	0.768	0.690	0.854	0.143	0.110	0.185	0.038	0.005	0.271

* Compared the hazard ratio (HR) of secondary knee procedures by graft type of reconstruction (autograft vs. allograft), gender (male vs. female), and age (<20, 20–39, 40–49, 50–59, ≥60).

**Table 4:** Follow-up time: index date~31 December 2021 (or death date).

	N	Follow-Up Time (Years)				
		Mean	s.d.	Q1 (p25)	Median	Q3 (p75)
Total	146,122	6.6	3.9	3.5	6.5	9.4
Autograft	24,974	7.4	4.1	4.2	7.5	10.3
Allograft	121,148	6.4	3.8	3.4	6.2	9.1

**Table 5:** Survival time (years): index date~outcome to occur.

	N	HTO				Menisectomy				Meniscus Repair				Meniscus Transplantation			
		N	Median	Mean	s.d.	N	Median	Mean	s.d.	N	Median	Mean	s.d.	N	Median	Mean	s.d.
Total	146,122	1073	4.3	5.1	3.9	15,218	2.1	3.3	3.1	7169	2.9	3.7	3.0	938	1.6	2.9	3.1
Autograft	24,974	119	6.1	6.0	4.1	2368	2.7	3.8	3.3	1255	3.2	4.2	3.2	192	1.6	3.2	3.6
Allograft	121,148	954	4.1	5.0	3.9	12,850	2.0	3.2	3.0	5914	2.9	3.6	3.0	746	1.6	2.9	3.0

## Data Availability

All data during this study are not publicly available because all data have been deposited in the National Health Insurance Service-HealthScreening (NHIS-HealS) Database. But all data during this study are included in this Article.

## References

[1] Gianotti S.M., Marshall S.W., Hume P.A., Bunt L. (2009). Incidence of anterior cruciate ligament injury and other knee ligament injuries: A national population-based study. J. Sci. Med. Sport.

[2] Janssen K.W., Orchard J.W., Driscoll T.R., van Mechelen W. (2012). High incidence and costs for anterior cruciate ligament reconstructions performed in Australia from 2003–2004 to 2007–2008: Time for an anterior cruciate ligament register by Scandinavian model?. Scand. J. Med. Sci. Sports.

[3] Sanders T.L., Maradit Kremers H., Bryan A.J., Larson D.R., Dahm D.L., Levy B.A., Stuart M.J., Krych A.J. (2016). Incidence of Anterior cruciate ligament tears and reconstruction: A 21-year population-Based study. Am. J. Sports Med..

[4] Longo U.G., Nagai K., Salvatore G., Cella E., Candela V., Cappelli F., Ciccozzi M., Denaro V. (2021). Epidemiology of Anterior Cruciate Ligament Reconstruction Surgery in Italy: A 15-Year Nationwide Registry Study. J. Clin. Med..

[5] Chung K.S., Kim J.H., Kong D.H., Park I., Kim J.G., Ha J.K. (2022). An Increasing Trend in the Number of Anterior Cruciate Ligament Reconstruction in Korea: A Nationwide Epidemiologic Study. Clin. Orthop. Surg..

[6] Meike E., Howell S.M., Hull M.L. (2017). Anterior laxity and patient-reported outcomes 7 years after ACL reconstruction with a fresh-frozen tibialis allograft. Knee Surg. Sports Traumatol. Arthroscop..

[7] Tashman S., Collon D., Anderson K., Kolowich P., Anderst W. (2004). Abnormal rotational knee motion during running after anterior cruciate ligament reconstruction. Am. J. Sports Med..

[8] Akesen B., Demirag B., Budak F. (2009). Evaluation of intra-articular collagenase, TIMP-1, and TNF-alpha levels before and after anterior cruciate ligament reconstruction. Acta Orthop. Traumatol. Turc..

[9] Tourville T.W., Johnson R.J., Slauterbeck J.R., Naud S., Beynnon B.D. (2013). Relationship between markers of type II collagen metabolism and tibiofemoral joint space width changes after ACL injury and reconstruction. Am. J. Sports Med..

[10] Chmielewski T.L., Trumble T.N., Joseph A.M., Shuster J., Indelicato P.A., Moser M.W., Cicuttini F., Leeuwenburgh C. (2012). Urinary CTX-II concentrations are elevated and associated with knee pain and function in subjects with ACL reconstruction. Osteoarthr. Cartil..

[11] Georgiev G.P., Yordanov Y., Olewnik K., Tubbs R.S., LaPrade R.F., Ananiev J., Slavchev S.A., Dimitrova I.N., Gaydarski L., Landzhov B. (2024). Do the Differences in the Epiligament of the Proximal and Distal Parts of the Anterior Cruciate Ligament Explain Their Different Healing Capacities? Quantitative and Immunohistochemical Analysis of CD34 and α-SMA Expression in Relation to the Epiligament Theor. Biomedicines.

[12] Jeon Y.S., Choi S.W., Park J.H., Yoon J.S., Shin J.S., Kim M.K. (2017). Mid-term outcomes of anterior cruciate ligament reconstruction with far anteromedial portal technique. Knee Surg. Relat. Res..

[13] Min J.H., Yoon H.K., Oh H.C., Youk T., Ha J.W., Park S.H. (2024). Graft choice to decrease the revision rate of anterior cruciate ligament reconstruction: A nationwide retrospective cohort study. Sci. Rep..

[14] Byun J., Yoon H.K., Oh H.C., Youk T., Ha J.W., Park S.H. (2024). Relationship between revision rate, osteoarthritis, and obesity for ACL reconstruction: A nationwide retrospective cohort study. Orthop. J. Sports Med..

[15] Cheung E.C., DiLallo M., Feeley B.T., Lansdown D.A. (2020). Osteoarthritis and ACL Reconstruction-Myths and Risks. Curr. Rev. Musculoskelet. Med..

[16] Risberg M.A., Oiestad B.E., Gunderson R., Aune A.K., Engebretsen L., Culvenor A., Holm I. (2016). Changes in Knee Osteoarthritis, Symptoms, and Function After Anterior Cruciate Ligament Reconstruction: A 20-Year Prospective Follow-up Study. Am. J. Sports Med..

[17] Lohmander L.S., Ostenberg A., Englund M., Roos H. (2004). High prevalence of knee osteoarthritis, pain, and functional limitations in female soccer players twelve years after anterior cruciate ligament injury. Arthrit. Rheumat..

[18] Kessler M.A., Behrend H., Henz S., Stutz G., Rukavina A., Kuster M.S. (2008). Function, osteoarthritis and activity after ACL-rupture: 11 years follow-up results of conservative versus reconstructive treatment. Knee Surg. Sports Traumatol. Arthroscop..

[19] Hui C., Salmon L.J., Kok A., Maeno S., Linklater J., Pinczewski L.A. (2011). Fifteen-year outcome of endoscopic anterior cruciate ligament reconstruction with patellar tendon autograft for “isolated” anterior cruciate ligament tear. Am. J. Sports Med..

[20] Oiestad B.E., Holm I., Aune A.K., Gunderson R., Myklebust G., Engebretsen L., Fosdahl M.A., Risberg M.A. (2010). Knee function and prevalence of knee osteoarthritis after anterior cruciate ligament reconstruction: A prospective study with 10 to 15 years of follow-up. Am. J. Sports Med..

[21] Oiestad B.E., Holm I., Engebretsen L., Risberg M.A. (2011). The association between radiographic knee osteoarthritis and knee symptoms, function and quality of life 10–15 years after anterior cruciate ligament reconstruction. Br. J. Sports Med..

[22] Cinque M.E., Chahla J., Mitchell J.J., Moatshe G., Pogorzelski J., Murphy C.P., Kennedy N.I., Godin J.A., LaPrade R.F. (2018). Influence of Meniscal and Chondral Lesions on Patient-Reported Outcomes After Primary Anterior Cruciate Ligament Reconstruction at 2-Year Follow-up. Orthop. J. Sports Med..

[23] Landsmeer M.L.A., Runhaar J., van Middelkoop M., Oei E.H.G., Schiphof D., Bindels P.J.E., Bierma-Zeinstra S.M.A. (2019). Predicting Knee Pain and Knee Osteoarthritis Among Overweight Women. J. Am. Board Fam. Med..

[24] Messier S.P., Mihalko S.L., Legault C., Miller G.D., Nicklas B.J., Devita P., Beavers D.P., Hunter D.J., Lyles M.F., Eckstein F. (2013). Effects of intensive diet and exercise on knee joint loads, inflammation, and clinical outcomes among overweight and obese adults with knee osteoarthritis: The IDEA randomized clinical trial. JAMA.

[25] Prodromos C., Joyce B., Shi K. (2007). A meta-analysis of stability of autografts compared to allografts after anterior cruciate ligament reconstruction. Knee Surg. Sports Traumatol. Arthroscop..

[26] Kraeutler M.J., Bravman J.T., McCarty E.C. (2013). Bone-patellar tendon-bone autograft versus allograft in outcomes of anterior cruciate ligament reconstruction: A meta-analysis of 5182 patients. Am. J. Sports Med..

[27] Lutz P.M., Feucht M.J., Wechselberger J., Rasper M., Petersen W., Wörtler K., Imhoff A.B., Achtnich A. (2021). Ultrasound-based examination of the medial ligament complex shows gender- and age-related differences in laxity. Knee Surg. Sports Traumatol. Arthroscop..

[28] Iriuchishima T., Shirakura K., Horaguchi T., Wada N., Sohmiya M., Tazawa F.M., Fu F.H. (2012). Age as a predictor of residual muscle weakness after anterior cruciate ligament reconstruction. Knee. Surg. Sports Traumatol. Arthroscop..

